# Colorectal Cancer Stem Cells and Targeted Agents

**DOI:** 10.3390/pharmaceutics15122763

**Published:** 2023-12-12

**Authors:** Haobin Zhao, Ruining Han, Zhankun Wang, Junfang Xian, Xiaosu Bai

**Affiliations:** 1 Department of General Practice, People’s Hospital of Longhua, 38 Jinglong Jianshe Road, Shenzhen 518109, China; 635274465@mail.nwpu.edu.cn (H.Z.); xianjunfang2022@163.com (J.X.); 2 Endocrinology Department, People’s Hospital of Longhua, 38 Jinglong Jianshe Road, Shenzhen 518109, China; 3 Obstetric Department, The Eighth Affiliated Hospital, Sun Yat-Sen University, Shenzhen 518033, China; guyuansiyu@163.com; 4 Emergency Department, People’s Hospital of Longhua, 38 Jinglong Jianshe Road, Shenzhen 518109, China; liuzhishusheng.001@163.com

**Keywords:** colorectal cancer stem cells, Lgr5, Wnt signaling pathway, single-cell omics technology

## Abstract

Since their discovery, cancer stem cells have become a hot topic in cancer therapy research. These cells possess stem cell-like self-renewal and differentiation capacities and are important factors that dominate cancer metastasis, therapy-resistance and recurrence. Worse, their inherent characteristics make them difficult to eliminate. Colorectal cancer is the third-most common cancer and the second leading cause of cancer death worldwide. Targeting colorectal cancer stem cells (CR-CSCs) can inhibit colorectal cancer metastasis, enhance therapeutic efficacy and reduce recurrence. Here, we introduced the origin, biomarker proteins, identification, cultivation and research techniques of CR-CSCs, and we summarized the signaling pathways that regulate the stemness of CR-CSCs, such as Wnt, JAK/STAT3, Notch and Hh signaling pathway. In addition to these, we also reviewed recent anti-CR-CSC drugs targeting signaling pathways, biomarkers and other regulators. These will help researchers gain insight into the current agents targeting to CR-CSCs, explore new cancer drugs and propose potential therapies.

## 1. Introduction

In 1994, John and Bonnet isolated and identified cancer cells with stemness from leukemia cells and proposed the concept of “leukemia stem cells (LSCs)” [1]. This was the first confirmation of the existence of stem cells in cancer, a major breakthrough in the field of cancer stem cell (CSC) research. In 2003, Dontu and colleagues isolated CSCs from breast cancer cells [2], providing the first proof of the existence of CSCs in solid tumors. In the following years, CSCs were found in brain tumors, prostate cancer, lung cancer, colorectal cancer and other tumors [3,4,5,6]. Nowadays, the theory of CSCs has gained consensus and has attracted much attention in cancer treatment research. CSCs are a small population of cancer cells with stemness like stem cells. They can achieve self-renewal through symmetrical division and asymmetric division to produce daughter cells with stemness or normal cancer cells [7]. Moreover, CSCs are capable of forming cancer cells with different degrees of differentiation and reassembling the complete cancer cell repertoire of the original cancer. In addition, normal cancer cells without CSC properties can dedifferentiate back into CSCs through a bidirectional interconversion process [8,9]. Normal cancer cells without CSC properties can dedifferentiate back into CSCs through a bidirectional interconversion process [10]. This is a major reason for cancer cell heterogeneity [11]. Cancer cells with or without CSC characteristics must be eradicated to achieve good therapeutic effects. During cancer development, CSCs are important factors that lead to metastasis, therapy-resistance and recurrence [12,13,14]. CSCs are often accompanied by an epithelial to mesenchymal transition phenotype, and they interact with stromal cells, endothelial cells and others to promote angiogenesis, promote stem-like cancer cell differentiation and accelerate metastasis [15]. The cell cycle of CSCs arrests in the G0 phase, so they are resistant to cycle specific chemotherapy drugs [16]. Due to their DNA synthesis asynchrony and enhanced DNA repair, CSCs are resistant to DNA damaging drugs [16]. Moreover, CSCs highly express drug transporters and anti-apoptotic proteins such as Bcl-2, which endows them with the ability to pump chemotherapy drugs out of the cell and resist programmed cell death [16]. Recent research has suggested that resting cancer stem cells can evade immune surveillance and lay the seeds for cancer recurrence [17,18]. This makes CSCs more difficult to eliminate than other cancer cells.

**Figure 1:**
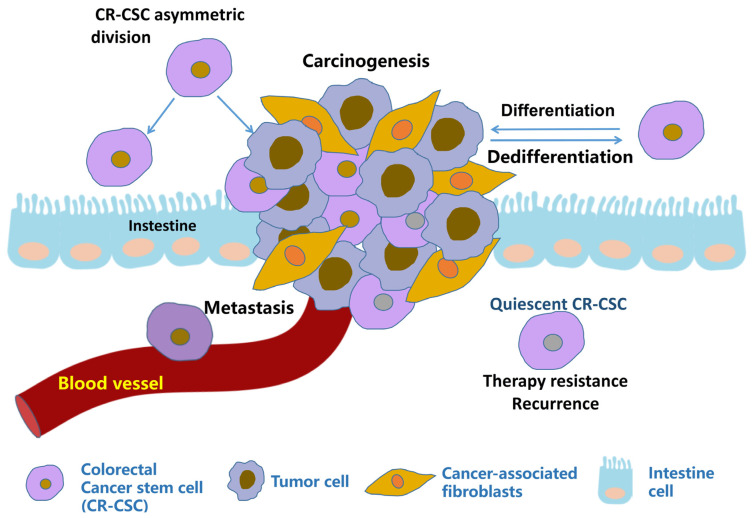
Illustration of metastasis, therapy resistance and recurrence promoted by CR-CSCs.

Colorectal cancer (CRC) is the third most common malignant tumor type. In recent years, with the popularization of early screening for colorectal cancer and the advancement of treatment methods, the mortality rate associated with colorectal cancer has decreased [19]. However, metastasis and recurrence are still the leading causes of death in most end-stage CRC patients. Reducing metastasis and recurrence remains an urgent problem in CRC therapy. Colorectal cancer stem cells (CR-CSCs) may be the initial cells of colon cancer [20], promoting colon cancer metastasis [21,22] and also one of the main culprits of therapy-resistance and recurrence [23] (Figure 1). Eliminating CR-CSCs can promote therapeutic effects against colon cancer [24,25,26]. Here, we reviewed the origin and identification of colorectal stem cells, and we summarized the potential therapeutic targets of CR-CSCs and the current research status of agents targeting CR-CSCs. This will help researchers to gain insight into the current agents targeting CR-CSCs, explore new drugs and propose potential therapies.

CR-CSCs not only divide into CR-CSCs, but can also produce ordinary cancer cells through proliferation or differentiation. Due to their quiescent state, high differentiation activity, secreting cytokines to make normal cells malignant and other properties, CR-CSCs can promote metastasis, therapeutic resistance and recurrence.

## 2. Colorectal Cancer Stem Cells

### 2.1. Origin of CR-CSCs

Researchers generally consider CSCs to have two main origins, derivation from normal cells that acquire mesenchymal properties [27] or transformation from normal adult stem cells [28]. The same holds true for the origin of CR-CSCs. In intestine, leucine-rich repeat-containing G-protein coupled receptor 5 (Lgr5) is expressed selectively in the crypt-base columnar cells [29] and was the first proven biomarker of CR-CSCs. In mouse models, genetic inactivation of the key colorectal cancer (CRC) driver gene Adenomatous Polyposis Coli (Apc) in Lgr5+ cells precipitated rapid tumor induction [30]. By downregulating β-Catenin and YAP signaling pathways, Protein kinase C ζ (PKC ζ) can inhibit intestinal stem cell function. PKC ζ deficiency can lead to an increase in stem cell activity in organoid cultures. Furthermore, tumorigenic activity increased in Lgr5+PKC ζ deficient mice [31]. This evidence suggests that CR-CSCs seem to originate from intestinal stem cells. However, selective and effective killing of Lgr5+ cells had no impact on primary tumor growth [24], and cells that disseminate and colonize distant organs were frequently Lgr5− [32]. Recent research using single cell sequencing technology has shown that the rDNA transcription and protein synthesis of Lgr5+ and Lgr5− cancer cell subsets were increased, which showed the characteristics of functional stem cells [33] and that lineage conversion between cell types can be driven by a combination of key CRC driver genes and microenvironmental extracellular signaling [34].Vazquez and colleagues also confirmed that the intestine contains two types of stem cells, Lgr5+ crypt-base columnar stem cells (CBCs) and Lgr5 regenerative stem cells (RSCs) using single cell sequencing technology. The two stem cell populations can coexist during tumorigenesis, exhibit dynamic plasticity, and complement each other to achieve homeostasis. The relative abundance of CBC-RSC is related to epithelial mutation and microenvironment signal destruction [35]. With the advancement of research technology, it is certain to uncover the origin of CR-CSCs.

### 2.2. Identification of CR-CSCs

**Figure 2:**
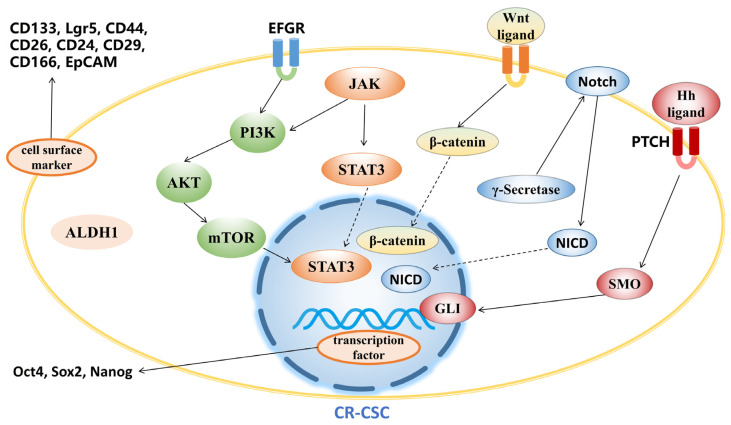
Biomarker proteins and regulators in pathways in CR-CSCs.

The sorting of cancer stem cells mainly relies on flow cytometry and magnetic activation sorting. The most commonly used basis is for sorting cancer stem cell biomarker proteins. Previous studies have found that CSCs have specific biomarkers, including CD133, ALDH1, CD44 and EpCAM [36]. CSC biomarkers vary with the tumor type. There are also some biomarkers for CR-CSCs. The marker proteins located on the cell membrane include Lgr5 [37], CD133 [38,39], CD44 [40], CD26 [41], CD24 [42], CD29 [43], CD166 [44] and EpCAM [45]. Aldehyde dehydrogenase1 (ALDH1) is an intracellular enzyme that oxidizes aldehydes and mediates the control of differentiation pathways. It is currently widely used as a marker for identifying and isolating various types of normal stem cells and CSCs [44,46]. Oct4 [47], Sox2 [48] and Nanog [49] are transcription factors used as biomarker located in the nucleus (Figure 2). The biological functions of most biomarkers are related to cell stemness. 

Biomarker proteins and regulators in the pathway are the most prominent targets in CR-CSC therapy.

By combining fluorescent labeled antibodies with cancer stem cell biomarkers, flow cytometry can be used to select CSCs expressing the related biomarkers from cancer cells. The side population (SP) cells with strong drug resistance are also considered to have the stemness of tumor stem cells. The characteristic of these cells is that they can expel the fluorescent dye hoechst33342 out of the cell, and it is shown as a non-fluorescent cell when detected via flow cytometry. CSCs with strong drug resistance in SP cells can be obtained by flow sorting [50]. Magnetic activated cell sorting utilizes antibodies attached to magnetic beads to bind to CSC biomarkers, adsorbing the corresponding cancer stem cells onto a separation column, while unbound cells pass through the separation column. Cancer stem cells with positive surface labeling can be obtained by mean of elution from the separation column [51,52] Single-cell omics technology is a powerful tool for exploring CSCs [53,54]. Single-cell omics technology can characterize and type CSCs in tumors, and establishing a stemness model has prospective clinical implications for prognostic evaluation [35,55].

### 2.3. Cultivation of CR-CSCs

It is worth emphasizing that although the research results on cancer stem cells have broad prospects for practical clinical applications, they are still in the initial stage. In order to successfully unleash the enormous potential of cancer stem cell research achievements, there are still many urgent issues to address. To understand the physiological activity of CSCs, the first step is to obtain them. For solid tumors, the most commonly used method to enrich cancer stem cells is non-adhesive culture with serum-free culture [56,57]. CSCs with self-renewal capacity are able to survive under non-adherent conditions and maintain clonogenic activity, whereas non-CSCs undergo anoikis by loss of anchorage.

Three-dimensional (3D) culture has emerged as a cell culture method in vitro in recent years. By using hydrogel to mimic the extracellular matrix and applying different culture conditions, 3D culture can mimic in vivo microenvironment [58]. Different gel materials have different porosity, permeability, surface chemical and mechanical properties, which will have different effects on cell growth and differentiation [59]. Three-dimensional culture can be used to enrich stem cells or study cell differentiation [60]. Organoid is an advanced version of 3D culture, which is a 3D micro cell cluster formed by directional differentiation of stem cells [61]. Organoids have the abilities to self-renew and self-organize, and can highly mimic the structure and function of organs in vivo. They have been widely used in the study of organ diseases, drug toxicity and cancer therapy [62,63].

## 3. Agents Targeting CR-CSCs

### 3.1. Targeting CR-CSC Biomarkers

Biomarker proteins are targets for the rapid screening of CRCs. In order to enhance the specificity of therapeutic strategies, researchers often choose ligands or antibodies against CSC surface makers (Table 1). MCLA-158 is an EGFR and Lgr5 targeting bispecific antibody with strong growth inhibitory effects on CRC organoids. Simultaneously, it exhibits strong anti-tumor activity in xenograft models derived from patients with high expression of Lgr5 and EGFR [64]. In mouse orthotopic xenograft models derived from CRC patients, MCLA-158 treatment not only reduced the size of the primary tumor but also effectively suppressed metastasis, including that of KRAS mutant tumors resistant to Cetuximab. Currently, researchers are conducting clinical trials of MCLA-158 in various solid tumors (NCT03526835) [64]. Catumaxomab was the first T cell binding bispecific antibody approved by the European Medicines Agency (EMA) in 2009 for the treatment of malignant ascites [65]. Catumaxomab is a trifunctional bispecific antibody that binds to EpCAM on cancer cells and CD3 on T cells. It also binds to FcγR to recruit immune helper cells [65]. Catumaxomab can effectively eliminate CD133+/EpCAM+CSCs in malignant ascites in patients with advanced ovarian cancer, gastric cancer and pancreatic cancer, which indicates that it has potential therapeutic applications in eradicating CSCs of epithelial cancers [66,67]. Similar to catumaxomab, solidomab is also a bispecific antibody targeting EpCAM and CD3. Solidomab treatment was found to effectively eradicated EpCAM+CSCs, originating from colon or pancreatic cancer patients that were inoculated into NOD/SCID mice [68,69]. 

In addition to antibodies, there are oncolytic virotherapies and CSC vaccines for targeted biomarker therapies. Oncolytic viruses are a class of viruses with tumor-killing functions. Oncolytic virotherapy is an emerging new tumor treatment that utilizes oncolytic viruses to selectively destroy tumor cells while leaving normal cells intact. Using the properties of oncolytic viruses combined with receptors on tumor cells, researchers have screened or engineered oncolytic viruses that target cancer stem cells [96]. Due to the characteristics of virus vectors, oncolytic virotherapy can trigger immunogenic cell death, release tumor-related antigens and elicit anti-tumor immune response, which can exert stronger anti-cancer effect [96]. Oncolytic viruses with a CD133-targeting motif effectively infected and killed CD133+CR-CSCs, and inhibited the growth of CRC xenotransplantation models [74]. Oncolytic virotherapy is one potential therapy strategy, but it still needs further research. CSCs vaccines are also a type of immunotherapy under research. For example, B16F10 CD133+/CD44+CSCs vaccine can effectively inhibit melanoma growth in mice and reduce the CSC population within tumors [97]. Although no cancer stem cell vaccine has entered clinical trials at this time, the demonstrated efficacy of a vaccine targeting metastatic CRC is reassuring and raises hope [98].

### 3.2. Targeting Signaling Pathway

Multiple signaling pathways are involved in the self-renewal, proliferation, apoptosis and angiogenesis processes of CR-CSCs. Currently, it is believed that specifically targeting cell signaling pathways to inhibit the effects of CR-CSCs is a major development direction for CRC therapy.

#### 3.2.1. Wnt Signaling Pathway

The Wnt pathway plays a critical role in controlling epithelial stem cell self-renewal, and its dysregulation causes colorectal carcinogenesis [99,100]. The canonical Wnt pathway downstream signaling is regulated by the level of β-catenin (Figure 2). TRAF2- and NCK-interacting kinase (TNIK) is an essential activator of Wnt target genes [99]. The inhibitory activity of TNIK inhibitors such as NCB0846 on CR-CSCs has been confirmed [75]. Epigallocatechin gallate (EGCG) is a kind of the catechins found in green tea. It has been proven to effectively inhibit stem cells from various cancers [101,102]. EGCG can inhibit the stemness of CRC cells by downregulating the expression of biomarkers such as CD133, CD44, NANOG, OCT4, ALDH1 and Wnt/β-catenin signaling pathway [76,77]. The small molecule inhibitor XAV939 was shown to significantly downregulate CSC biomarkers in colon cancer cells and increased apoptosis induced by chemotherapy drugs [78]. Phenethyl isothiocyanate (PEITC) and sulforaphane are natural products extracted from cruciferae plants with anti-cancer activities [79,103]. PEITC suppressed the characteristics of CR-CSCs by reducing the activity of the Wnt/β-catenin pathway, leading to a decline in the proportion of CD133+ cells [79,80]. Salinomycin, an anti-bacterial polyether isolated from Streptomyces albus, was found to selectively eliminate CD133+ cells in CRC [104]. Salinomycin induced apoptosis of human CR-CSCs by activating caspase, increasing DNA damage and disrupting of the Wnt/β-catenin/TCF complex. Tumor growth and expression of CSC-related Wnt genes, including Lgr5 were decreased [82,105]. In addition to these, there are many drugs that reduce CSC stemness by targeting the Wnt signaling pathway, such as pan-inhibitor of histone demethylases JIB04 [83] and lysine-specific demethylase 1 inhibitor CBB1003 [84] (Table 1).

#### 3.2.2. Hedgehog Signaling Pathway

The Hedgehog (Hh) signaling pathway plays an essential role in the growth and differentiation of gastrointestinal tissue [106]. The canonical Hh signal involves Hh ligands (sonic Hh, Indian Hh or desert Hh) binding to the patched (PTCH) receptor, releasing smoothened (SMO) and causing the receptor to activate. In this process, GLI protein will be activated and become transcriptional activators of the downstream targets of the Hh signaling pathway. The Hh-GLI pathway is involved in maintaining the self-renewal ability of CR-CSCs [107,108] (Figure 2).

Vismodegib (also named Ericdge, GDC-0449) is a Hedgehog signaling pathway inhibitor used in clinical practice and approved by the US Food and Drug Administration for the treatment of basal cell carcinoma. Vismodegib targets a subpopulation of CSCs in basal cell carcinoma [109]. Studies have shown that vismodegib can inhibit the stemness of CR-CSC and the expression of biomarkers CD44 and ALDH1 [110]. Cyclopamine is a natural alkaloid that can inhibit the Hh-GLI signaling pathway by inhibiting SMO. After cyclopamine treatment, the mRNA levels of CSC biomarkers and genes related to Hh signaling, including PTCH1, SMO and GLI1 were found to decreased in stem cells derived from HCT116 [111]. Given the regulation of CR-CSCs by Hh signaling pathway, more new inhibitors are being developed (Table 2).

#### 3.2.3. Notch Signaling Pathway

Notch signaling is involved in the regulation of cell differentiation, proliferation and tumorigenesis [134]. The pathway consists of four receptors (Notch1-4) and five ligands (Jagged-1, Jagged-2, Delta-1, Delta-3, Delta-4) and DNA-binding proteins. The interaction between receptors and ligands initiates protein cleavage cascade reactions, leading to the activation of Notch target genes [135]. Gamma secretase inhibitors (GSIs) can inhibit Notch signaling by preventing the proteolytic cleavage of Notch receptors [136] (Figure 2). However, RO4929097, one of the GSIs, failed to achieve excellent results in clinical trials [113]. More GSIs are under investigation. DLL4 is an activator protein of the non-canonical Notch signaling pathway.DLL4 antibody was confirmed to be effective against both KRAS wild-type and mutant CRC cells, effectively eradicating CR-CSCs and enhancing the antitumor effect of irinotecan [114,137]. In addition, Honokiol, Quercetin and others have also been shown to have the ability to inhibit CR-CSC stemness [115,116] (Table 2).

#### 3.2.4. PI3K/Akt/mTOR Signaling Pathway

The PI3K/Akt/mTOR signaling pathway plays a crucial role in cell metabolism and proliferation, and it is closely related to the CR-CSC phenotype [138]. Studies have demonstrated that components of the PI3K/Akt signaling pathway are overexpressed in CRC in vitro and in vivo [130,139]. PI3K and MEK inhibitors used in combination can induce CR-CSC death and the regression of tumor xenografts [140]. BEZ235, a dual pathway inhibitor of mTOR and PI3K, could inhibit the proliferation of CR-CSCs and the expression of its biomarkers CD133 and Lgr5, thus suppressing the stemness of CR-CSCs [118]. LY294002 is a PI3K inhibitor based on the flavonoid quercetin. LY294002 blocked Akt phosphorylation through the PI3K/Akt signaling pathway and inhibited liver CSC proliferation and tumorigenicity in vitro and in vivo [120]. LY294002 treatment led to a decrease in proliferation, spheroid formation and self-renewal properties, as well as a decrease in Akt phosphorylation and cyclin D1 expression in CR-CSCs in vitro [120]. Piplartine is an alkaloid amide isolated from peppers. It was reported to inhibit stemness properties in leukemia and oral cancer [121,140]. In combination with auranofin, piplartine reduced the expression levels of surface biomarker CD44v9, eliminated CR-CSCs and inhibited CRC growth [121]. Rapamycin is an mTOR inhibitor and is used clinically as an immunosuppressive drug. In CRC cell lines, it has the potential to decrease the spheroid-forming ability and ALDH1 activity [123]. In cotreatment with 5-FU and oxaliplatin, rapamycin reduced the CR-CSCs subpopulation. Metformin is also reported to reduce the CSC population in different types of cancers [141]. Metformin not only reduced the proliferation of CSC population in mouse xenografts [125], but also effectively reduced CSC population in colorectal and other gastrointestinal cancers in a pilot clinical trial [142]. There are also many drugs that target the PI3K/Akt/mTOR signaling pathway to inhibit CR-CSCs, such as Atractylenolide I and Torin-1 [126,127].

#### 3.2.5. JAK/STAT3 Signaling Pathway

JAK/STAT signaling is closely related to cancer growth and metastasis. In cancer cells, JAK/STAT signaling can be activated by multiple mechanisms, most notably by STAT3 activation [143]. High STAT3 activity was found in CRC-SCs, but not in normal colon epithelial cells [144]. Another study revealed that the JAK2/STAT3 signaling pathway promoted the persistence and radio-resistance of CR-CSCs [145]. Curcumin is a polyphenol from Curcuma longa, and GO-Y030 is a novel curcumin analog. Curcumin and its analog GO-Y030 were proposed drug candidates to eliminate CR-CSCs by suppressing STAT3 activity [132]. Napabucasin, also named BBI608, is an orally administered STAT3 inhibitor with anti-CSC activity against various types of cancer [146,147]. However, unfortunately, napabucasin failed to achieve satisfactory results in phase 3 clinical trials for the treatment of colorectal cancer [133]. ls. Napabucasin may be the first anti-CRC drug approved for clinical use targeting CSCs

There are other signaling pathways such as TGF-β and Hippo, regulating CSCs stemness. These various signaling pathways do not operate independently and often act via crosstalk to influence cancer progression [22,106,148,149,150] (Table 2).

### 3.3. Other Agents Targeting CR-CSCs

FBXL5 E3 ligase plays an important role in maintaining the stemness of CR-CSCs. The anandamide uptake inhibitor AM404 can suppress FBXL5 expression and inhibit CR-CSC dedifferentiation, migration and drug resistance [151]. Prexasertib, also named LY2606368, is an investigational checkpoint kinase inhibitor. By inhibiting checkpoint kinase (CHK) 1, LY2606368 affected DNA replication in most CR-CSCs [152]. ASR352 and NSC30049 are both CHK1 inhibitor [153,154]. RAB5/7, which is associated with the endo lysosomal pathway, plays an important role in the survival and maintenance of CSCs through the mitophagic pathway. Mefloquine, an anti-malaria drug, has been identified as a new inhibitor of RAB. In the PDX model of colorectal cancer, mefloquine can target RAB5/7 to inhibit the mitophagic pathway and induce mitochondrial-induced apoptosis, thereby exerting anti-tumor effects without significant side effects [155]. At present, there are many other types of CR-CSC antagonists, such as pitavastatin [156], histone deacetylase inhibitor trichostatin A [157] and inhibitors of the post-translational sumoylation modification pathway [158]. They may play an important role in targeting CR-CSCs in future (Table 3).

## 4. Future Prospects

Despite significant progress in research on therapeutic drugs for CR-CSCs, cancer treatment still faces many challenges. Tumor microenvironment (TME) plays a major role in determining cell fate and behavioral choices [165,166]. Under the complex interaction of the TME, reversible transformation can be achieved between tumorigenic and non-tumorigenic cells. This is the reason why it is difficult to completely remove CSCs [167]. Cancer-associated fibroblasts (CAFs) play a significant positive role in the development and transfer of CR-CSCs [168]. A tumor is an entity composed of multiple heterogeneous cells. Different subtypes of CSCs may have different resistance mechanisms, and therefore, each cancer subtype may require unique therapies [169]. The plethora of contributing factors in cancer and the complex regulatory network make it difficult to eradicate cancer via a single therapeutic intervention.

Fortunately, researchers never give up. In order to achieve effective treatment, more extensive and in-depth research has been conducted to examine molecular and cellular aspects, including the synergistic targeting of CR-CSCs and TME in cancer treatment. Fibroblast activation protein (FAP) is a type II membrane-bound glycoprotein that is overexpressed in CAFs and activated fibroblasts at wound healing/inflammatory sites. FAP inhibitor has been developed to target CAFs to improve TME [170]. In response to the problem of tumor stem cell heterogeneity, anti CSC drugs with diverse targets have been or are currently being developed. Many of them have been incorporated into clinical or preclinical trials. In the face of the differing responses of different patients to therapeutic approaches, prognosis prediction and personalized treatment are the best solutions. Single cell omics and organoid technology can assist in achieving this goal. Using large-scale omics technologies, we can subtype cancers and build predictive models for treatment response [35,55]. In vitro culture of patient derived tumor organoids can enable prediction of drug sensitivity and resistance, and achieve precision treatment [171]. In summary, in the face of differing treatment responses in patients, the heterogeneity of cancer stem cells and the complex regulatory mechanisms of cancer, researchers have been struggling to decipher them.

## 5. Conclusions

CR-CSCs are a small group of stem cells in colon cancer that have unlimited proliferation, self-renewal and differentiation ability, playing an important role in drug resistance, metastasis and recurrence. CSCs are like cancer seeds, which cannot be ignored in cancer treatment. The advancement of modern medical technology has given us a certain level of understanding of colon cancer stem cells, but we have not yet fully understood them. Regarding the current situation of CR-CSCs targeted inhibitors, it is important to strengthen the synergistic effect between drugs. By combining drugs targeting CR-CSCs with other treatment methods, we can prevent cancer metastasis and recurrence while reducing the occurrence of drug resistance, which will improve the effectiveness of current CRC treatment. Cancer and the tissue involved are integrated, and treatment should adopt a systematic approach, striving to completely eliminate the seeds to prevent metastasis and recurrence. Targeted inhibitors of CRCSCs are an emerging treatment method for CRC. Although there are still many unclear mechanisms to be discovered, it can be expected that in the future, these drugs will play an undeniable role in preventing colon cancer metastasis and recurrence. Certainly, a complete cancer treatment requires not only targeted treatment for CR-CSCs, but also targeted combination therapy for non-CR-CSCs and TME, as well as the entire tumor. In order to benefit all patients, personalized therapy is the ultimate goal. Single-cell omics technology and organoid technology have contributed to a deeper understanding of the different aspects of cancer stem cells and to the development of more effective treatments for cancer. Achieving this goal still requires considerable efforts and collaboration from researchers.

## Figures and Tables

**Table 1:** Agents targeting to CR-CSC biomarkers and Wnt pathway.

Agents	Targets of CR-CSCs	Efficacy	References
MCLA-158	EFGR and Lgr5	Effective in preclinical models	[64]
Catumaxomab	EpCAM	Approved in the European Union for the treatment of malignant ascites	[65,66,67]
Solidomab	EpCAM	Effective in vitro	[68,69]
CD133-directed CAR T cells	CD133	Effective in a phase I trial	[70]
Cetuximab	EFGR	Effective in combination therapies	[71,72,73]
CD133-targeted oncolyticvirus	CD133	Effective in mice	[74]
NCB0846	Wnt pathway	Effective in mice	[75]
Epigallocatechin gallate	Wnt pathway	Effective in mice	[76,77]
XAV939	Wnt pathway	Effective in vitro	[78]
Phenethyl isothiocyanate and sulforaphane	Wnt pathway	Not proven effective in trials	[79,80,81]
Salinomycin	Wnt pathway	Effective in mice	[82]
JIB04	Wnt pathway	Effective in mice	[83]
CBB1003	Wnt pathway	Effective in vitro	[84]
YW2065	Wnt pathway	Effective in mice	[85]
LF3	Wnt pathway	Effective in mice	[86]
Dickkopf-2	Wnt pathway	Effective in vitro	[87]
ICG-001	Wnt pathway	Effective in vitro	[88]
4-Acetyl-antroquinonol B	Wnt pathway and JAK-STAT pathway	Effective in mice	[89,90]
Diallyl trisulfide	Wnt pathway	Effective in vitro	[91]
36-077	Wnt pathway	Effective in vitro	[92]
Evodiamine	Wnt and Notch pathway	Effective in vitro	[93]
Farnesyl dimethyl chromanol	Wnt pathway	Effective in mice	[94]
FH535	Wnt pathway	Effective in vitro	[95]

**Table 2:** Agents targeting to signaling pathway.

Agents	Targets of CR-CSCs	Efficacy	Reference
Vismodegib	SMO of Hedgehog pathway	Approved by FDA for the treatment of basal cell carcinoma	[110,112]
Cyclopamine	SMO of Hedgehog pathway	Effective in vitro	[111]
RO4929097	γ-secretase of Notch pathway	Not proven effective in a phase II trial	[113]
Anti-DLL4	DLL4 of Notch pathway	Effective in a phase I trial	[114]
Honokiol	γ-secretase of Notch pathway	Effective in mice	[115]
Quercetin	γ-secretase of Notch pathway	Effective in mice	[116]
α-Mangostine	Notch pathway	Effective in vitro	[117]
BEZ235	PI3K/Akt/mTOR pathway	Not proven effective in a phase Ib trial	[118,119]
LY294002	PI3K/Akt/mTOR pathway	Effective in vitro	[120]
Piplartine	PI3K/Akt/mTOR pathway	Not proven effective in trials	[121,122]
Rapamycin	mTOR of PI3K/Akt/mTOR pathway	Not proven effective in trials	[123,124]
Metformin	mTOR of PI3K/Akt/mTOR pathway	Effective in combination therapies	[125]
Atractylenolide I	PI3K/Akt/mTOR pathway	Effective in mice	[126]
Torin-1	PI3K/Akt/mTOR pathway	Effective in vitro	[127]
Buparlisib	Akt of PI3K/Akt/mTOR pathway	Effective in a phase Ib trial	[128,129]
MK-2206	Akt of PI3K/Akt/mTOR pathway	Not proven effective in a phase II trial	[130,131]
Curcumin and GO-Y030	STAT3 of JAK/STAT3 signaling pathway	Effective in mice	[132]
Napabucasin	STAT3 of JAK/STAT3 signaling pathway	Not proven effective in a phase III trial	[133]

**Table 3:** Agents targeting CR-CSCs.

Agents	Targets of CR-CSCs	Efficacy	Reference
AM404	FBXL5	Effective in mice	[151]
LY2606368	Checkpoint kinase 1	Effective in a phase II trial of ovarian cancer	[152,159]
ASR352	Checkpoint kinase 1	Effective in vitro	[153]
NCS30049	Checkpoint kinase 1	Effective in vitro	[154]
Mefloquine	RAB5/7	Effective in vitro	[155]
Pitavastatin	——	Effective in vitro	[156]
Trichostatin A	histone deacetylase	Effective in vitro	[157]
Dabrafenib	BRAF	Approved by FDA for the treatment of elanoma	[160]
Mithramycin A	SP1	Effective in vitro	[161]
Parthenolide	USP47	Effective in vitro	[162]
Gambogic acid	ZFP36	Effective in a phase IIa trial	[163,164]

## Data Availability

Not Applicable.
